# The Compatibility of Three Silicone Oils with Polydimethylsiloxane and the Microstructure and Properties of Their Composite Coatings

**DOI:** 10.3390/polym13142355

**Published:** 2021-07-18

**Authors:** Yuguo Jiang, Zhanping Zhang, Yuhong Qi

**Affiliations:** Department of Materials Science and Engineering, Dalian Maritime University, Dalian 116026, China; 1120180332jyg@dlmu.edu.cn (Y.J.); yuhong_qi@dlmu.edu.cn (Y.Q.)

**Keywords:** silicone, coating, silicone oil, compatibility, polydimethylsiloxane

## Abstract

The compatibility of three types of silicone oil with polydimethylsiloxane, the phase separation of their mixture and the microstructure and properties of their composite coatings were investigated. The existing form of silicone oil in the coating and the precipitation behavior were also studied. The compatibility observed experimentally of the three silicone oils with PDMS is consistent with the results of the thermodynamic calculation. The silicone oil droplet produced by phase separation in the mixture solution can keep its shape in the cured coating, also affecting the microstructure and mechanical properties of the coating. It was found that methyl silicone oil and methyl fluoro silicone oil do not precipitate on the surface, and they have no effect on the surface properties of the coating. In contrast, phenyl silicone oil has obvious effect on the surface, which makes the water contact angle and diiodomethane contact angle of the coating decrease significantly.

## 1. Introduction

Marine biological fouling is one of the most common fouling forms in the ocean [1,2], which has caused great harm and potential safety hazard to ships, bridges, offshore drilling platforms and other offshore industrial facilities [3,4]. It is an effective and long-standing method to an apply antifouling coating on the surface of the metal immersed in seawater that needs to be protected [5]. It can not only inhibit the attachment of marine pollutants such as algae, oysters and even tiny bacteria and reduce the harm of marine biological pollution and but also effectively improve the life of ships and industrial facilities [3,6]. Organotin self-polishing antifouling coatings were widely used because of their excellent properties [7]. However, research found that the organotin antifouling coatings in use caused pollution to the ocean, damaging the marine ecological environment. With the promulgation and implementation of the “International convention for the control of harmful antifouling systems on ships” (AFC), many countries have turned to study environmentally friendly marine antifouling coatings [8,9].

Silicone coatings can effectively prevent fouling organisms from adhering to the coating surface [10]. Due to its low surface energy and low elastic modulus, most of the mucus secreted by fouling organisms cannot wet the coating surface, meaning it is difficult for it to be fixed on the coating surface [11,12]. The lower elastic modulus enables the fouling organisms on the coating surface to peel off from the surface with lower energy under the scouring of seawater [13], which can effectively remove the attached organisms by the scouring effect of the ship and seawater during navigation [14], which is an efficient “fouling release” coating [15].

Organosilicon was first used in marine antifouling coatings in 1972, developed by Mueller [16]. However, it was not widely used due to the shortcomings of its poor mechanical properties, construction and recoating property. After 1990, Yonehara and colleagues produced a series of organosilicon coatings with different compositions and additives. Through panel immersion tests, they proved that the antifouling effect of silicone antifouling coatings was improved compared with traditional antifouling coatings [17]. After that, Masato, Takafumi and colleagues made different modifications to silicone antifouling coatings and achieved a good antifouling effect [18,19]. Ba et al. improved the mechanical properties of silicone coatings by adding different nano-powders [20].

Ships that stay in the port for a long time tend to collect and deface organisms on the surface [21]. The antifouling properties are often improved by adding silicone oil to silicone coatings to mimic the properties of silicone oil deposited on the skins of large animals in the ocean [22,23]. Moreover, the low toxicity of silicone oil poses little risk to marine life [24]. The earliest study on silicone oil-modified silicone coatings was in 1977; Milne tried to add inert silicone oil into silicone coatings to improve the antifouling performance [25]. Hoipkemeier-Wilson et al. added methyl silicone oil to silicone coatings and proved that adding silicone oil improved the antifouling performance through a spore adhesion experiment [26]. Truby et al. added phenyl silicone oil to silicone coatings and conducted long-term panel immersion tests in Hawaii; the results showed that the addition of silicone oil effectively reduced the adhesion strength of barnacles and oysters to the silicone coating [27].

During recent years, some research progress has been made on adding silicone oil to improve antifouling performance [28,29,30]. Research on mixing silicone oil into silicone coatings mainly focuses on the antifouling performance of some specific fouling organisms [31,32]. Ba et al. further expounded the influence of the type, viscosity and addition amount of silicone oil on the precipitation property of silicone oil on the surface [33]. Related studies were mainly focused on the antifouling performance of silicone coatings. However, the influence of the compatibility of silicone oil and silicone blend solutions on the structure of the blend coating is still unknown. This paper will focus on comparing the compatibility of three types of silicone oil with PDMS through thermodynamic calculation and contrast observations of the blend solution, and the influence of the compatibility on the mechanical properties and surface properties of the composite coatings containing solid and liquid phases.

## 2. Materials and Methods

### 2.1. Material

Hydroxy-terminated polydimethylsiloxane (PDMS) was purchased from Shandong Dayi Chemical Industry Co., Ltd. (Yantai, China), with a viscosity of 10,000 Pa·s and molecular weight of 60,000. Methyl silicone oil (MSO) was purchased from Shandong Dayi Chemical Industry Co., Ltd. (Yantai, China), with a viscosity of 15 Pa·s and a molecular weight of 1700. Phenyl silicone oil (PSO) was purchased from Shanghai Hualing Resin Co., Ltd. (Shanghai, China), with a viscosity of 30 Pa·s and a molecular weight of 450. Methyl fluoro silicone oil (FSO) was purchased from Wuhan Huaxiang Kejie Biotechnology Co., Ltd. (Wuhan, China), with a viscosity of 1000 Pa·s and a molecular weight of 26,000. Ethyl orthosilicate (TEOS) was obtained from Tianjin Chemical Co., Ltd. (Tianjin, China). New bismuth decanoate (BIND) was obtained from Shanghai Deyin Chemical Co., Ltd. (Shanghai, China). Xylene and ethyl acetate were also analytical grade and provided by Yongda Chemical Reagents Co., Ltd. (Tianjin, China).

### 2.2. Preparation of Coating Sample

Generally, silicone paint consists of three parts: film former, crosslinking agent and catalyst. The film former used in the experiment is hydroxyl-terminated PDMS, which needs to be modified by adding silicone oil (silicone oil does not participate in the film-forming reaction).

Silicone paint with silicone oil was prepared by a two-step method: First, 200 g PDMS, and silicone oil with the corresponding mass ratio were added into a 500 mL metal stirring tank. The mixture was dispersed for 30 min at 3000 rpm by using a sand mill dispersion mixer. After the dispersion, the mixture was poured into a clean tinplate tank for standing and defoaming to form component A. TEOS and xylene were mixed into a solution according to the mass ratio of 3:7, poured into a clean white plastic bottle and recorded as component B. BIND and ethyl acetate were mixed into a solution according to the mass ratio of 3:7 and poured into a clean white plastic bottle, which was marked as component C. The three components, A, B and C, were mixed according to the mass ratio of 20 (PDMS): 4:1 and stirred with a paint mixing knife for 3 min, followed by painting and curing to obtain the composite coating.

The blank control sample without PSO was set as P0. The coating samples were set as X-ZY, in which the letter X is the type of silicone oil, where MSO represents methyl silicone oil (MSO), PSO represents phenyl silicone oil (PSO) and FSO represents fluoro silicone oil (FSO); the letter Z is the ratio (O/B) of silicone oil to base material, and the letter Y represents the mass of silicone oil per 100 g of PDMS.

### 2.3. Characterization

#### 2.3.1. Phase Separation of Silicone Oil/PDMS Blend Solution

PDMS was blended with silicone oil at a mass ratio of 10%, 20%, 30% or 40% to 90%. After dispersing for 10 min with a multi-purpose mixer at the speed of 3000 rpm, the mixture was poured into the test tube and kept for 48 h. The phase separation state was observed and recorded by taking a photo. The blend solution samples were set as X%Y/PDMS, in which the letter X is the mass of silicone oil per 100 g of PDMS, and the letter Y is the type of silicone oil, where MSO represents methyl silicone oil (MSO), PSO represents phenyl silicone oil (PSO) and FSO represents fluoro silicone oil (FSO).

#### 2.3.2. Microscopic Observation of Silicone Oil/PDMS Blend Solution

A small amount of the prepared mixed solution absorbed by the pipette was dripped on the glass slide with about 0.2 g solution, which was allowed to stand for 48 h to make the mixed solution fully spread on the surface of the glass slide. Then, the sample was observed with an optical microscope (OM), and photos were taken. The mixture samples were set as XY, in which the letter X is the type of silicone oil, where MSO represents methyl silicone oil (MSO), PSO represents phenyl silicone oil (PSO) and FSO represents fluoro silicone oil (FSO); the letter Y is the mass of silicone oil per 100 g of PDMS.

#### 2.3.3. Fracture Observation of the Coatings

The coating was cut into 40 × 10 mm^2^ samples. They were immersed in liquid nitrogen and cooled for 20 min before breaking. The fracture morphology was observed by an Olympus OLS4000 confocal laser scanning microscope (CLSM) (Olympus (China) Co, Ltd, Beijing, China).

#### 2.3.4. Microscopic Surface Characterization 

The surface of the coatings with different silicone oils was observed by a WT-1000GM stereomicroscope (Shanghai micro path photoelectric technology, Shanghai, China) and by CLSM, photographed. The photos were processed by Photoshop software (Adobe Systems, San Francisco, CA, USA) to obtain the ratio of the silicone oil coverage area and coating coverage area.

#### 2.3.5. Mechanical Properties of the Coatings

The tensile curve of the coating was measured using a UTM5105 computer-controlled electronic universal testing machine (Jinan Wance Testing Electric Equipment Co., Ltd., Jinan, China), with a tensile rate of 50 mm/min. The dumbbell-shaped sample was prepared according to the national standard, GB/T 528-2009 (ISO 37:2005 [34]); the length of the sample was 75 mm, the gauge length was 25 mm and the width was 4 mm. The tensile data less than 0.1 mm/mm were selected to fit the elastic modulus of the coating. Three samples were prepared and tested for each coating. The stress–strain curve was plotted.

The sample was placed on the surface of the operating table, and the TH2200 shore hardness tester (Beijing Shidai Zhifeng Technology Co., Ltd. Beijing, China) was held to make the presser foot parallel to the surface of the sample. The presser needle was pressed into the sample vertically without vibration. When the hardness tester made contact with the sample surface, it read and recorded the data within 1 s, selecting 6 points for each sample, taking the average value as the hardness. To meet the test requirements, the thickness of the sample should be more than 5 mm.

#### 2.3.6. Surface Properties of Coatings

The contact angles of the coatings were measured by the angle measuring method. A JC2000C contact angle measuring instrument (China Zhongchen Co., Ltd., Shanghai, China) was used to extrude about 3 μL of distilled H_2_O and CH_2_I_2_, and the camera of the equipment was used to save the image and record the outline of the contact angle between the droplet and the surface. For every coating, five positions were measured, and the average value was taken as the contact angle. Based on the measured water and diiodomethane contact angle (CA), the surface energy of the sample was calculated by the Owens double liquid method.

## 3. Results

### 3.1. Phase Separation Thermodynamic of Silicone Oil/PDMS Mixture

**Figure 1:**
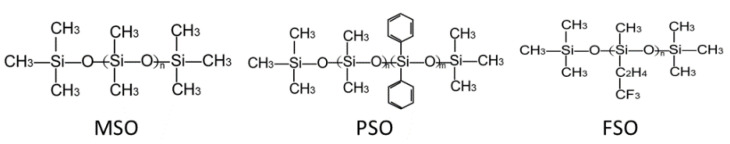
The structural formula of three types of inert silicone oils.

The chemical structures of three types of silicone oils, MSO, PSO and FSO, are shown in Figure 1. The different chemical structures determine their difference with PDMS, and the different compatibilities of each silicone oil and PDMS mixture.

The Flory–Huggins lattice model is most commonly used to describe the mixing phenomenon of polymer solutions. Based on this model [35,36], therefore, the thermodynamics of the silicone oil/PDMS blend solutions can be analyzed as follows.

Taking silicone oil as polymer A and PDMS as polymer B, we state that the molecular chain of polymers A and B contains “chain segments” x_A_ and x_B_, respectively. The amount of each substance in the silicone oil/PDMS mixture is n_A_ and n_B_, and the volume fraction is φ_A_ and φ_B_, respectively. The molar volume of both “chain segments” A and B is Vu. The total volume of the mixture system is V. χ_1_ is the interaction parameter between the silicone oil and PDMS. The unit mixed Gibbs free energy ∆G_M_ of the mixture can be calculated by formula (1) [35].

∆G_M_ = R T V (φ_A_ lnφ_A_/x_A_ + φ_B_ lnφ_B_/x_B_ + χ_1_ φ_A_ φ_B_)/Vu (1)

For a blend solution, we usually observe the phase separation point of the blend solution by an observation method and then substitute the data into Equation (1) to obtain the interaction parameters of the blend solution. However, by combining thermodynamics with the Flory–Huggins lattice model, we obtain the relationship between the interaction parameter χ_1_ and the solubility parameter of silicone oil δ_1_ and PDMS δ_2_; V_M1_ is the molar volume of silicone oil [36].

Χ_1_ = V_M1_(δ_1_ − δ_2_)^2^/RT (2)

The solubility parameter of PDMS δ_2_ was determined by the intrinsic viscosity method in this investigation, and it is equal to 15 (J/cm^3^)^1/2^. Therefore, the solubility parameter of the silicone oil δ_1_ obtained by the group contribution method was substituted into Equation (2), and the interaction parameter χ_1_ between silicone oil and PDMS was obtained. As the main chain structure of silicone oil and PDMS is the same, their repeating unit is a Si-O bond, the repeating unit can be regarded as a “chain segment” and the “chain segment” number of the polymer can be estimated by the relative molecular weight of the polymer. The molecular weight of PDMS resin is 60,000, and the number of chain segments is 810. The thermodynamic parameters calculated are listed in Table 1.

**Figure 2:**
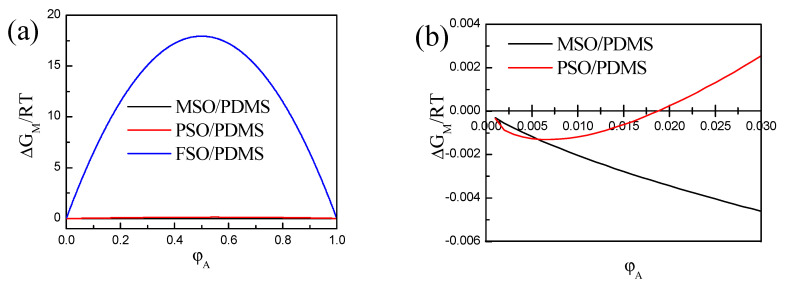
Mixed Gibbs free energy of three silicone oil with PDMS blends: (**a**) whole concentration range; (**b**) local amplification for low concentrations.

Substituting the data in Table 1 into Equation (1), the curves of the concentration φ_A_ and ∆G_M_ of the mixture system of silicone oil and PDMS were obtained, as shown in Figure 2. According to thermodynamics, when ∆G_M_ < 0, the blend solution can spontaneously fuse, and when ∆G_M_ > 0, phase separation will inevitably occur. As it is shown in Figure 2a, for the mixed solution of methyl fluoro silicone oil and PDMS at any concentration, ∆G_M_ > 0, and this means that methyl fluoro silicone oil and PDMS cannot be miscible, and phase separation between methyl fluoro silicone oil and PDMS is inevitable. On the contrary, as shown in Figure 2a,b, when methyl silicone oil and PDMS are blended, ∆G_M_ is always less than 0 at any ratio, that is to say, methyl silicone oil and PDMS are miscible, and phase separation cannot occur. As it is shown in Figure 2b, when the concentration of phenyl silicone oil and PDMS is low, ∆G_M_ < 0, and then it is more than 1.9%, ∆G_M_ > 0, which means that when the volume concentration of phenyl silicone oil reaches 1.9%, phase separation begins to appear. In other words, the larger the interaction parameter χ_1_ between silicone oil and polydimethylsiloxane, the lower the compatibility between silicone oil and polydimethylsiloxane, and the easier it is for phase separation to occur.

### 3.2. Experimental Verification of Silicone Oil/PDMS Phase Separation

**Figure 3:**
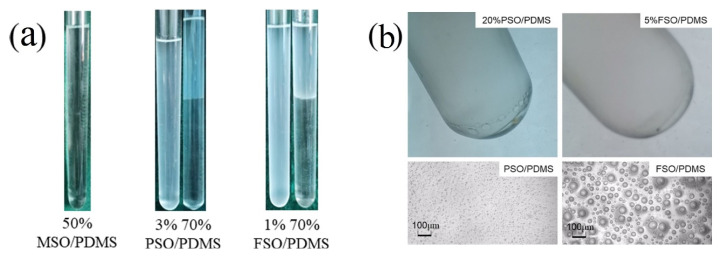
Morphology of different silicone oil/PDMS mixtures: (**a**) macroscopic images; (**b**) OM images.

The experimental observation, as shown in Figure 3, verified the validity of the above-mentioned thermodynamic analysis. The structure of methyl silicone oil is similar to that of PDMS, but the difference lies in the groups of the end groups. In any proportion of the blend solution, methyl silicone oil and PDMS were miscible, as shown in Figure 3a, and no phase separation was observed; therefore, methyl silicone oil and PDMS are completely compatible.

The structural difference between phenyl silicone oil and PDMS mainly comes from the benzene ring on the side chain, which leads to the poor compatibility between phenyl silicone oil and PDMS. When the concentration was 1%, the mixed solution in the test tube was transparent. When the concentration was 2%, it became turbid. When the concentration was 3%, the mixed solution remained turbid, as shown in Figure 3a. With the increase, precipitated phenyl silicone oil droplets were observed at the bottom at a 20% concentration, as shown in Figure 3b, and an obvious stratification phenomenon was observed when the PSO concentration was up to and over 30%. After standing for half an hour, the delamination was completed and could maintain stability, as shown in Figure 3a.

There are perfluorinated groups in the side chain of FSO. Due to the polarity of fluorine atoms, the compatibility between FSO and PDMS is very poor. FSO has the worst compatibility, and phase separation even occurs at very low concentrations. As it is shown in Figure 3a, when the concentration is 1%, a turbid mixture can be observed. When the concentration was 5%, as shown in Figure 3b, FSO droplets precipitated at the bottom of the test tube. At up to 10%, the phenomenon of delamination was obviously observed. However, because of the high viscosity of FSO, the intermolecular movement is slow. Although it can be layered at a low concentration, it often takes several hours to complete the delamination. 

**Figure 4:**
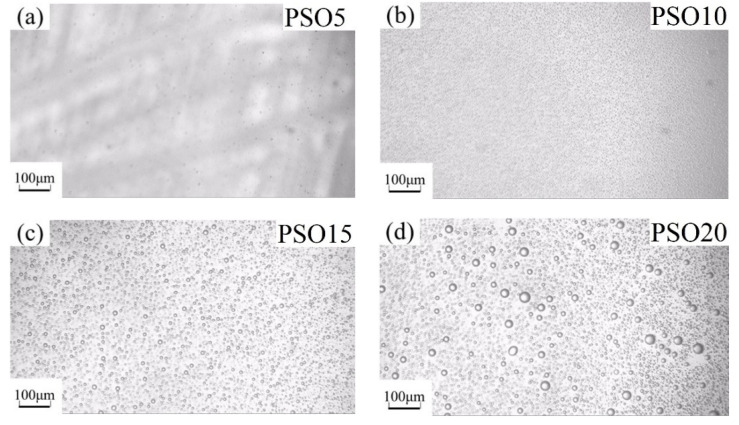
OM images of PSO/PDMS mixtures: (**a**) PSO5, (**b**) PSO10, (**c**) PSO15, (**d**) PSO20.

**Figure 5:**
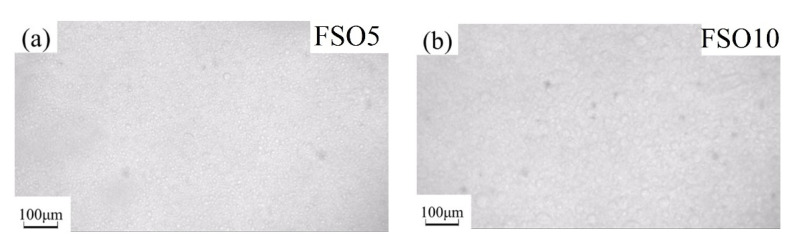

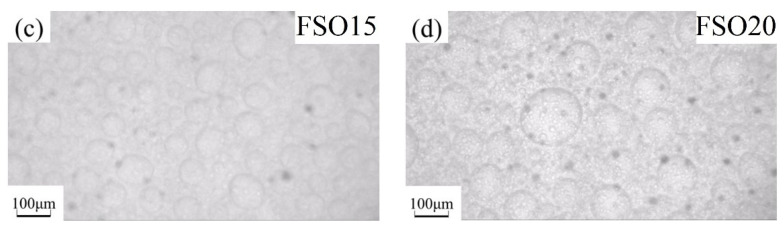
OM images of FSO/PDMS mixtures: (**a**) FSO5, (**b**) FSO10, (**c**) FSO15, (**d**) FSO20.

The silicone oil droplets changed when increasing silicone oil’s concentration in PDMS, which is shown, respectively, for PSO/PDMS and FSO/PDMS mixtures in Figure 4 and Figure 5. The immiscible silicone oil droplets can be seen clearly in the blend solution under the microscope. Furthermore, the size of the droplets became larger after increasing the concentration of silicone oil. The size of FSO droplets was larger than that of PSO droplets, due to the weaker compatibility of FSO with PDMS. 

**Figure 6:**
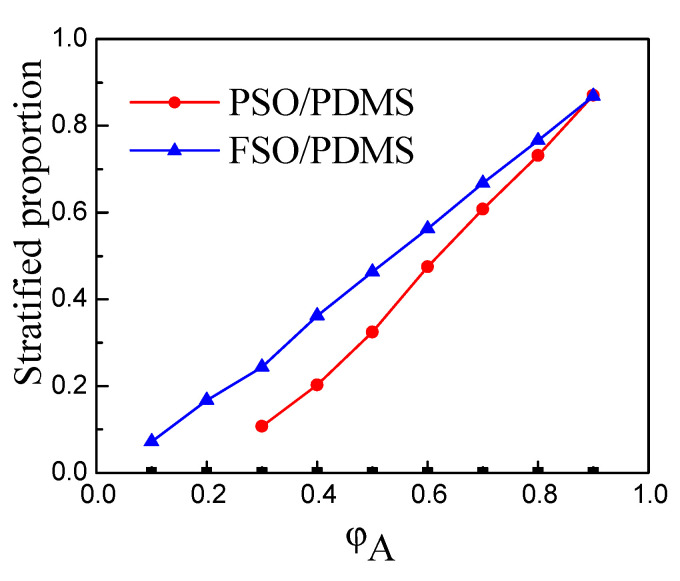
Concentration of silicone oil and stratified proportion.

Further quantitative analysis shows that the proportion of layered silicone oil in the total volume of the mixture increases linearly with the increase in the silicone oil concentration, as shown in Figure 6, and the limit proportion of the two types of PSO and FSO tends to be the same.

### 3.3. Fracture Microstructure of the Cured Coatings

**Figure 7:**
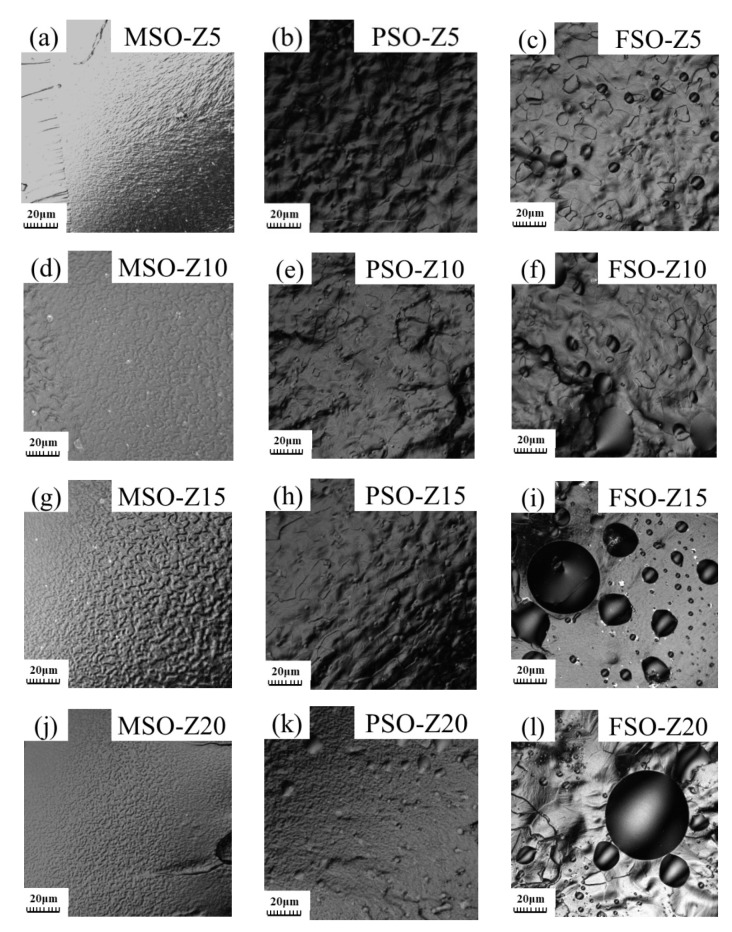
CLSM fracture morphology of different coatings: (**a**) MSO-Z5, (**b**)PSO-Z5, (**c**) FSO-Z5, (**d**)MSO-Z10, (**e**) PSO-Z10, (**f**) FSO-Z10, (**g**) MSO-Z15, (**h**) PSO-Z15, (**i**) FSO-Z15, (**j**) MSO-Z20, (**k**) PSO-Z20, (**l**) FSO-Z20.

The compatibility of silicone oil and polydimethylsiloxane and the degree of phase separation in the mixed solution will inevitably affect the microstructure of the cured coating. As it is shown in Figure 7a,d,g,j, the MSO droplets produced by phase separation cannot be found in the fracture of samples MSO-Z5, MSO-Z10, MSO-Z15 and MSO-Z20. The solubility of MSO and PDMS is excellent, and they can dissolve each other in any proportion. In the liquid state, the phase separation of MSO dissolved in PDMS still did not occur during the film-forming process.

The trace of PSO droplets on the fracture surface of the coating can be found in Figure 7b,e,h,k. 

The compatibility between FSO and PDMS is very bad. The phase-separated FSO droplets can be seen clearly in Figure 7c,f,i,l. Compared with PSO, FSO has poor miscibility with PDMS and high viscosity, meaning the volume of FSO droplets formed under the same stirring action was large. Therefore, the size of FSO droplets on the fracture is bigger than that of PSO.

When comparing Figure 4 with Figure 5 and Figure 7, it can be found that when the phase-separated silicone oil can be observed in the blending solution, the phase-separated silicone oil can also be observed at the fracture. When the volume of phase-separated silicone oil is large, the large and irregular silicone oil droplets can also be observed at the fracture; this shows that the free silicone oil droplets in the coating are related to the silicone oil droplets in the blend solution, and the silicone oil droplets in the blend solution will continue to exist in the silicone coating in a free form during the curing process.

### 3.4. Leaching Behavior of Silicone Oil on the Coating

**Figure 8:**
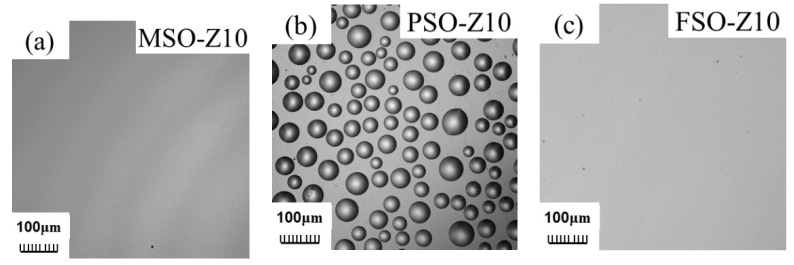
CLSM morphology of silicone oil/PDMS coatings: (**a**) MSO-Z10, (**b**) PSO-Z10, (**c**) FSO-Z10.

**Figure 9:**
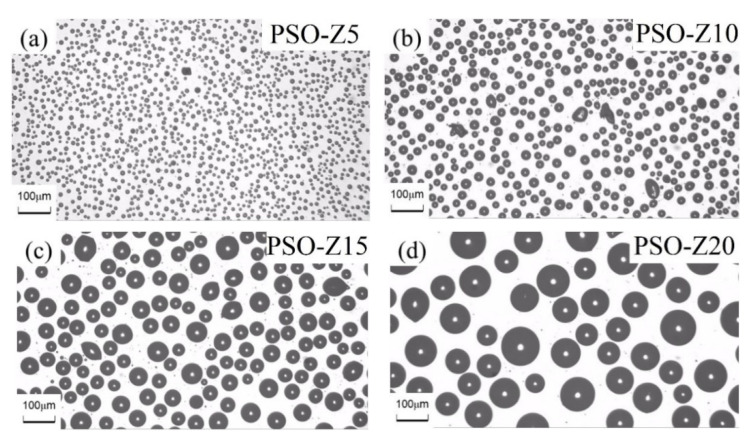
OM morphology of leached PSO droplets on PSO/PDMS coatings: (**a**) PSO-Z5, (**b**) PSO-Z10, (**c**) PSO-Z15, (**d**) PSO-Z20.

The morphologies of the three types of coatings exposed for 1 month to ambient conditions were observed by OM and CLSM, shown in Figure 8. As it is shown in Figure 8a,c, both MSO and FSO cannot be observed even by CLSM on the coating surface. There are no free silicone oil droplets in the coating, meaning there is no silicone oil precipitation on the coating surface. As MSO is miscible with PDMS in any proportion, phase separation cannot occur between MSO and PDMS. On the contrary, the surface of the FSO/PDMS coating has no leached trace of silicone oil due to the poor compatibility of FSO with PDMS, and to the higher density of FSO. As it is shown in Figure 8b, phase-separated PSO droplets can be observed. The reason is that PSO is partially compatible with PDMS, which can diffuse through capillary action and move to the coating surface. With the increase in the PSO content in PDMS, as shown in Figure 9, not only the coverage area of leached PSO on the coating surface increased, where it increased from 28.09% for coating PSO-Z5 to 37.23% for PSO-Z10, 43.36% for PSO-Z15 and 46.04% for PSO-Z20, but also the size of the leached PSO droplets increased.

### 3.5. Mechanical Properties of the Composite Coatings

**Figure 10:**
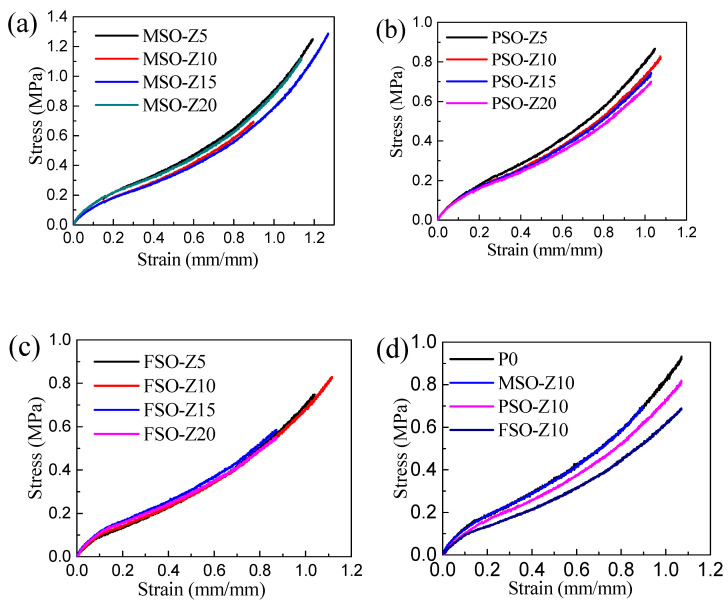
Tensile stress–strain curves of silicone coatings: (**a**) MSO/PDMS coating, (**b**) PSO/PDMS coating, (**c**) FSO/PDMS coating, (**d**) different silicone oil/PDMS coatings.

**Figure 11:**
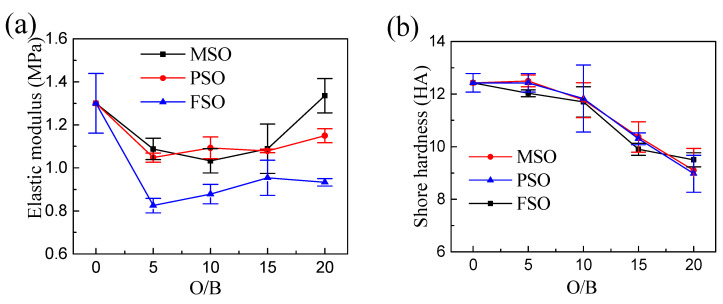
Mechanical properties of coatings change with O/B in the coatings of different silicone oils: (**a**) elastic modulus, (**b**) hardness.

The tensile stress–strain curves of the coatings are shown in Figure 10. For the same silicone oil, the stress–strain curves and tensile behavior of the coatings hardly changed when increasing the silicone oil concentration. Compared to the pure PDMS coating, as shown in Figure 10d, the tensile curve of MSO-Z10 is highly coincident with PDMS because methyl silicone oil is dissolved in the silicone coating, and there is no phase separation. However, under the same strain, the tensile stress of PSO-Z10 and FSO-Z10 decreased to some degree due to phase separation, which resulted in silicone oil droplets forming in the coating. The droplets formed in FSO-Z10 are larger and more severe than those in PSO-Z10. Therefore, the decline in the mechanical properties is more serious. In addition, by introducing silicone oil, as shown in Figure 11, the elastic modulus and hardness of the coating decreased to a certain extent respectively with the increase in the silicone oil addition. In conclusion, the lower the compatibility of silicone oil with PDMS, the more silicone oil droplets produced by phase separation during the curing process of the coating, the larger the size of the droplets and the lower the elastic modulus and hardness of the composite coating.

### 3.6. Surface Energy of the Composite Coatings

**Figure 12:**
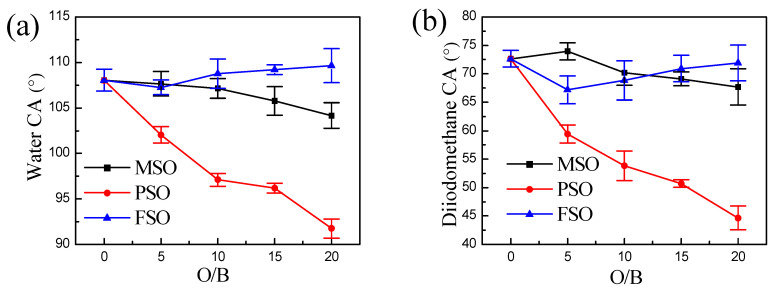
CA changes with O/B in the coatings of different silicone oils, (**a**) water contact angle and (**b**) diiodomethane contact angle.

For the coating with MSO, there is no MSO precipitation on the surface. The structure and group of MSO and PDMS are similar. Therefore, it can be seen from Table 2 and Figure 12 that the surface energy increases slightly with the addition of methyl silicone oil. However, the water and diiodomethane contact angle do not change obviously with increasing MSO, which indicates that the addition of MSO has little effect on the surface properties of the coating.

However, a large amount of silicone oil was precipitated on the surface of the PDMS coating with the added PSO. As shown in Figure 12, the higher the amount of PSO, the higher the amount of PSO that is precipitated on the coating. Compared with PDMS, PSO has many phenyls in the side chain, and phenyl has polarity. Therefore, the water contact angle and diiodomethane contact angle of the coating decreased significantly with the increase in PSO, but the surface energy of the coating increased.

The side chain of FSO has a perfluorinated group, which has strong polarity and an amphiphilic property. However, no FSO precipitates on the surface of the coating. Therefore, both the contact angle and surface energy hardly changed with the increase in FSO.

## 4. Conclusions

The phase separation of MSO, PSO and FSO with PDMS blend solutions was consistent with the thermodynamic calculation. When the concentration of PSO and FSO reaches a certain degree, the delamination phenomenon will appear.

The distribution of silicone oil in the blend coating was the same as that in the blend solution. The morphology of silicone oil droplets produced by phase separation in the blend solution was consistent with that observed in the fracture surface of the coating, which indicates that the phase-separated droplets in the blend solution were fixed during the curing process and remained free.

There was no silicone oil on the surface of the MSO/PDMS coating and FSO/PDMS coating. In contrast, silicone oil could be observed on the surface of the PSO/PDMS coating, and the amount of silicone oil precipitation increased with the increase in the PSO content.

The mechanical properties of the coating decreased with the increase in the silicone oil content. There was no silicone oil on the surface of the MSO/PDMS coating and FSO/PDMS coating after being exposed for 1 month to ambient conditions. The water contact angle, diiodomethane contact angle and surface energy hardly changed. PSO was easy to move onto the surface of the composite coating. The contact angle and surface energy of the coating obviously reduced with increasing PSO content.

Therefore, phenyl silicone oil is the best choice among the three silicone oils to improve the antifouling performance by imitating the behavior of marine organisms secreting mucus. The leaching effect of PSO can be controlled by its content and compatibility with PDMS.

## Figures and Tables

**Table 1:** The thermodynamic parameters of silicone oil/PDMS.

Types of Silicone Oil	MSO	PSO	FSO
χ_1_	0.0074	0.8118	71.7798
δ_1_/(J/cm^3^)^1/2^	15.10	17.22	15.83
x_A_	22	6	351
V_M1_/(mL/mol)	1770.83	432.69	17,894.00

**Table 2:** The surface properties of different silicone oil/silicone coatings.

Sample	Contact Angle (°)		Surface Free Energy (mJ/m^2^)
	Water	Diiodomethane	
P0	108.05 ± 1.21	72.65 ± 1.49	21.49 ± 0.86
MSO-Z10	107.15 ± 1.09	70.15 ± 1.52	22.89 ± 0.86
PSO-Z10	97.10 ± 0.51	53.85 ± 1.83	32.39 ± 1.02
FSO-Z10	108.75 ± 1.15	68.85 ± 2.43	23.79 ± 1.41

## Data Availability

Data are contained within the article.
